# Case report: Single gene testing and comprehensive genomic profiling in non-small cell lung cancer—a case series of divergent results from a large reference laboratory

**DOI:** 10.3389/fonc.2024.1445668

**Published:** 2024-10-31

**Authors:** Kyle C. Strickland, Mary K. Nesline, Rebecca A. Previs, Heidi Ko, Maureen Cooper, Jennifer R. Rushton, Zachary D. Wallen, Sarabjot Pabla, Jeffrey M. Conroy, Mark Sausen, Kamal S. Saini, Luca Cantini, Taylor J. Jensen, Brian J. Caveney, Marcia Eisenberg, Eric A. Severson, Shakti Ramkissoon

**Affiliations:** ^1^ Labcorp Oncology, Durham, NC, United States; ^2^ Duke University Medical Center, Duke Cancer Institute, Department of Pathology, Durham, NC, United States; ^3^ Duke University Medical Center, Duke Cancer Institute, Department of Obstetrics & Gynecology, Division of Gynecologic Oncology, Durham, NC, United States; ^4^ Baptist Health System, San Antonio, TX, United States; ^5^ Labcorp Oncology, Buffalo, NY, United States; ^6^ Labcorp Oncology, Baltimore, MD, United States; ^7^ Fortrea Inc., Durham, NC, United States; ^8^ Addenbrooke’s Hospital, Cambridge University Hospitals NHS Foundation Trust, Cambridge, United Kingdom; ^9^ Wake Forest Comprehensive Cancer Center and Department of Pathology, Wake Forest School of Medicine, Winston-Salem, NC, United States

**Keywords:** non-small cell lung cancer (NSCLC) molecular testing, single gene testing (SGT), comprehensive genomic profiling (CGP), next-generation sequencing (NGS), cancer genomics

## Abstract

Clinical management of non-small cell lung cancer (NSCLC) requires accurate identification of tumor-specific genetic alterations to inform treatment options. Historically, providers have relied on single-gene testing (SGT) for actionable variants due to a perception of cost-effectiveness and/or efficient turnaround time compared to next-generation sequencing (NGS). However, not all actionable variants may be evaluated through SGT modalities, and an SGT approach can exhaust valuable tissue needed for more comprehensive analyses. In contrast, comprehensive genomic profiling (CGP) tests employ NGS to sequence megabases of DNA and RNA to evaluate all relevant molecular alterations, providing a broader genetic profile to identify actionable alterations that SGT may not accurately or efficiently assess. Here, we briefly describe four cases from a large reference laboratory in which actionable alterations were identified by CGP but not SGT. The discussion highlights the utility and advantages of using CGP to provide complete and timely treatment options and clinical trial opportunities for patients with NSCLC.

## Introduction

The treatment paradigm of advanced non-small cell lung cancer (NSCLC) has shifted from a generalized approach using cytotoxic chemotherapy to a more targeted approach using therapies prescribed on the basis of specific genomic alterations, which has necessitated the clinical integration of precision medicine principles (1). NSCLC accounts for approximately 85% of all lung cancer diagnoses (2),, and introduces complex therapeutic challenges due to pathologic and molecular heterogeneity. The development of an ever-increasing array of complex biomarker-directed single agent and combination therapies requires accurate, timely, and broad molecular testing. Comprehensive genomic profiling (CGP) techniques have the potential to address many challenges of large-scale genomic testing, due to its ability to efficiently assess a vast array of genetic alterations through massively parallel sequencing (3).

Despite the promises and performance of CGP, providers continue to utilize single gene testing (SGT) methodologies to identify actionable mutations in the advanced/metastatic disease setting, driven by perceptions of cost-effectiveness for patients and relatively rapid turnaround time (4, 5). However, SGT has some important shortcomings, such as the inability to identify certain molecular alterations due to inherent sequencing limitations and a restricted scope of detectable mutations (6, 7), as well as an increased likelihood of exhausting finite tissue samples that may be needed for additional testing (8). In contrast, a comprehensive approach to molecular profiling examines a broad spectrum of actionable targets in a single assay that conserves tissue, without sacrificing sensitivity or specificity.

In this case series, we describe four NSCLC patient cases for which initial SGT provided divergent or incomplete results compared to subsequent CGP. Each case illustrates tangible advantages to ordering CGP over SGT in the clinical setting. We discuss the practical implications of these findings and examine the advantages of CGP in the context of the current literature concerning NSCLC precision medicine and molecular profiling. Additionally, we consider how divergent SGT and CGP results may impact the NSCLC patient and their general perspective of their medical care.

## Methods

We obtained approval for this study from an external institutional review board, the Western Institutional Review Board Copernicus Group (WCG protocol #1340120). Using data from the Labcorp^®^ reference laboratory information system, we identified providers in the United States who ordered SGT for patients with advanced/metastatic NSCLC during standard care and offered no-cost CGP testing for their patients in addition to SGT (if found to be negative) or instead of SGT altogether. All SGT and CGP tests were completed by December 2022, with final reports delivered to ordering providers following standard procedure.

All SGT and CGP were performed at a Labcorp^®^ testing facility. SGT included assays of formalin-fixed paraffin-embedded (FFPE) tissue to detect clinically actionable biomarkers, including: fluorescent *in situ* hybridization (FISH) to detect rearrangements in *ALK*, *RET*, and/or *ROS1*, SNaPshot multiplex polymerase chain reaction (PCR) to detect pathogenic variants in *BRAF*, *KRAS*, and/or *EGFR*, and immunohistochemistry (IHC) to evaluate PD-L1 expression (**Supplementary Table 1**). CGP was performed using OmniSeq^®^ INSIGHT (Labcorp Oncology, Buffalo, NY), an assay that utilizes the TruSight^®^ Oncology 500 panel (Illumina, San Diego, CA), as previously described (9). In this assay, DNA and RNA is extracted from FFPE tissue, and DNA is sequenced to detect small nucleotide variants, insertions, and deletions in the full coding regions of 523 genes, as well as to evaluate 59 genes for copy number alterations. Additionally, RNA is sequenced to detect fusions and splice site variants in 55 genes, and PD-L1 expression (22C3 antibody) was also performed. DNA/RNA sequencing and IHC results were all included in a single integrated report.

## Case descriptions

Ordering providers, including oncologists and pathologists from 80 community practices, participated in the study and ordered CGP for a total of 561 patients with advanced/metastatic NSCLC, of which 150 (27%) had negative prior SGT results (5). Of these cases with negative SGT results, we identified 4 patients (2.6%) that were negative for highly actionable genomic variants by SGT but demonstrated positive actionable findings by CGP, either in the same gene or for a different gene.

**Figure 1:**
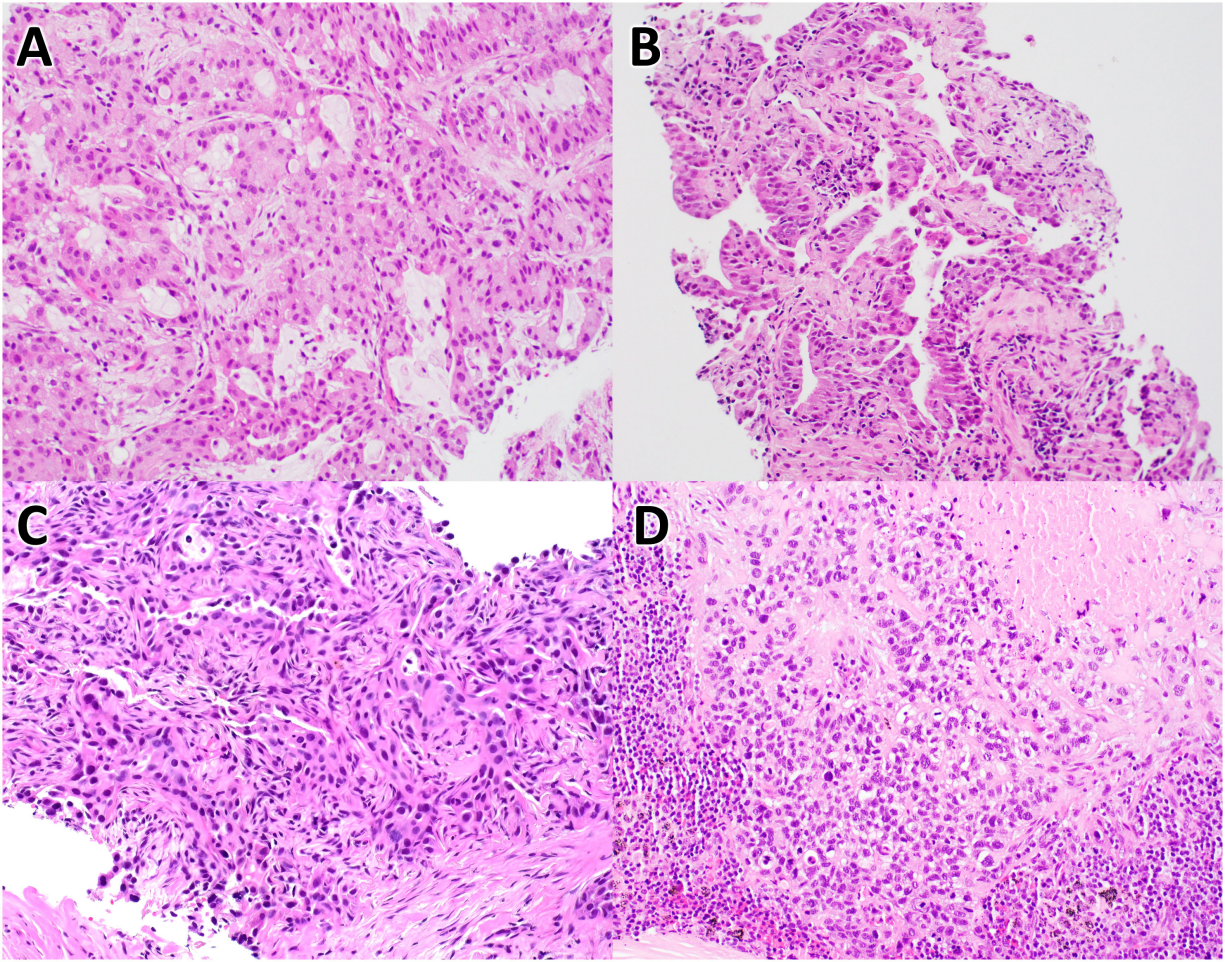
Histology of non-small cell lung carcinoma cases. These images are of H&E stained tissues sections from case #1 **(A)**, case #2 **(B)**, case #3 **(C)**, and case #4 **(D)**, at 20X magnification.

Case #1: A woman in her early 40’s with a history of gastrointestinal and uterine cancers was found to have a lung mass in the right upper lobe. A CT-guided core needle biopsy revealed moderately differentiated adenocarcinoma (**Figure 1A**). IHC was positive for CK-7, TTF-1, and Napsin A, but negative for CK20 and CDX2, consistent with a primary lung adenocarcinoma. The initial molecular analysis (reported 24 days post-biopsy) aimed at identifying alterations by SGT, which included FISH for *ROS1* and *ALK* rearrangements and SNaPshot multiplex PCR to detect *EGFR* variants, all of which were negative for actionable alterations. In contrast, CGP (ordered 67 days after collection) was subsequently performed and identified two *ALK* fusions, *EML4-ALK* (EML4:chr2:in6:42492089 and ALK:chr2:ex20:29446394) and *ALK-MAP4K3* (ALK:chr2:ex13:29462607 and MAP4k3:chr2:in1:39664031), as well as an *ALK* SNV of G1202R (**Table 1**). CGP also reported the tumor as microsatellite stable, with a low TMB of 2.3 mut/Mb. PD-L1 testing, initially performed alongside SGT and later with CGP, showed a high tumor proportion score (TPS) of 70% initially and later evaluated as 20%.

**Table 1:** Cases with divergent single gene and next generation sequencing test results.

Biomarker	SGT Method	Case 1 Results		Case 2 Results		Case 3 Results		Case 4 Results	
		SGT	CGIP	SGT	CGIP	SGT	CGIP	SGT	CGIP
Time from Sample Collection to Sample Received (Days)	n/a	11	67	5	120	3	233	5	11
Turnaround Time from Sample Received to Report (Days)	n/a	7	7	13	6	13	9	20	7
*ALK*	*ALK* Gene Rearrangements by FISH	**negative**	**ALK-MAP4K3 and EML4-ALK fusions**	negative	negative	negative	negative	n/r	negative
*MET*	*C-MET* Oncology FISH for Amplification	n/r	negative	n/r	negative	**Deletion of MET and likely polysomy 7**	**MET exon 14 skipping (c.2942-14_2942-1delinsA)**	negative	negative
*RET*	*RET* Gene Rearrangements by FISH	n/r	negative	negative	negative	negative	negative	negative	negative
*ROS1*	*ROS1* Gene Rearrangements by FISH	negative	negative	negative	negative	negative	negative	negative	negative
*BRAF*	SNaPshot Multiplex PCR (primer extension-based method)	n/r	negative	negative	negative	negative	negative	**n/r**	**V600E**
*EGFR*	SNaPshot Multiplex PCR (primer extension-based method)	negative	negative	**negative**	**D770_771insG**	negative	negative	n/r	negative
*KRAS*	SNaPshot Multiplex PCR (primer extension-based method)	n/r	negative	negative	negative	negative	negative	n/r	Q61R
MSI	n/a	n/r	MSS	n/r	MSS	n/r	MSS	n/r	MSS
PD-L1 IHC	IHC	positive	positive	positive	positive	positive	positive	positive	positive
PD-L1 TPS	IHC	70%	20%	90%	50%	10%	10%	60%	95%
TMB (mut/Mb)	n/a	n/a	2.3	n/a	6.2	n/a	1.5	n/a	19.5
Additional CGIP Results	n/a	n/a	n/a	n/a	*BRCA1* truncation (S713*), *DHX15* truncation (Q136*), *PIK3CA* SNV (H1047R)	n/a	*ARID2* truncation (Q284*)	n/a	*BCOR* frameshift (F646fs, K1417fs), *NOTCH1* SNV (Q61R), *NRAS* SNV (Q61R), *PMS2* frameshift (L729fs), *PTEN* SNV (c.802-1G>T), *TP53* SNV (E285K)
Same Tissue Block Used for SGT and CGIP? (Yes/No)	n/a	Yes		Yes		Yes		No	

Pertinent results are highlighted in bold red text. Regarding SGTs methodologies: FISH was used to evaluate *ALK*, *MET*, *RET*, and *ROS1* and conventional sequencing was used to evaluate *BRAF*, *EGFR*, and *KRAS*.

CGIP comprehensive genomic and immune profiling; IHC immunohistochemistry; n/r not reported; n/a not applicable; SGT single gene testing; TMB tumor mutational burden; TPS tumor proportion score.

Case #2: An man in his early 80’s with a left lung mass underwent core needle biopsy to reveal a moderately differentiated adenocarcinoma with papillary features (**Figure 1B**). Per report, IHC showed positive TTF1 and focal p40 staining, consistent with a primary lung adenocarcinoma. Initial testing by SGT (ordered 18 days after submission) included FISH for *ALK*, *RET*, and *ROS1* rearrangements and SNaPshot multiplex PCR to detect *BRAF*, *EGFR*, and *KRAS* variants. CGP was conducted 126 days post-biopsy and identified an *EGFR* exon 20 insertion, along with other SNVs not detected by SGT (**Table 1**). CGP analysis also determined the tumor to be microsatellite stable with a low TMB of 6.2 mut/Mb. PD-L1 was tested on two occasions, both with positive results (90% initially as SGT, later reported with CGP as 50%).

Case #3: A woman in her mid-70’s with a 2.9 cm mass in the left upper lung lobe underwent a lobectomy, which demonstrated a lepidic predominant, moderately differentiated adenocarcinoma (**Figure 1C**). IHC on the surgical specimen showed positivity for CK7, TTF1, and Napsin A, and negative staining for CK20, consistent with primary lung adenocarcinoma. Initial SGT, reported 15 days post-surgery, included FISH analysis to detect rearrangements in *ALK*, *RET*, and *ROS1* and amplification of MET, in addition to SNaPshot multiplex PCR to detect *BRAF*, *EGFR*, and *KRAS* variants. FISH for *MET* did reveal loss of *MET* in a subset of cells, as well as additional copies of *MET* and the control probe, consistent with polysomy of chromosome 7. All other SGTs were negative for actionable variants. Subsequent CGP, performed 242 days post-surgery on archival tissue, identified a *MET* exon 14 skipping alteration (**Table 1**), a finding not detected by earlier SGT for *MET* amplification. Additionally, PD-L1 immunohistochemistry, conducted at diagnosis alongside SGT and again with CGP, showed two consistent TPS results (10%).

Case #4: A male in his late 60’s with mediastinal lymphadenopathy underwent testing on two different tumor samples. Per report, an initial biopsy revealed a non-small cell lung carcinoma, squamous cell type, and SGTs were performed on this sample (ordered 25 days post-collection), including FISH for *MET* and *ROS1*, which were negative for alterations. Subsequently, additional tissue as part of mediastinal lymph node sampling, which was performed 34 days after the initial collection. Histologic evaluation revealed metastatic poorly differentiated squamous cell carcinoma with necrosis involving two of nine lymph nodes (**Figure 1D**). CGP was ordered 11 days after this second sample and identified a *BRAF* V600E mutation, as well as other genetic alterations (**Table 1**). CGP also demonstrated a high TMB (19.5 mut/Mb) and stable microsatellite sites. PD-L1 IHC, performed initially and alongside CGP, showed positive results in both cases (60% in the right lung biopsy sample, 95% in the subsequent lymph node sample).

## Discussion

This case series illustrates important limitations of SGT in the management of advanced NSCLC, particularly in comparison to the performance of CGP. Each case shows the utility of CGP to broadly identify actionable alterations that may otherwise be missed by workflows relying on SGT alone. The adoption of precision medicine for the treatment of NSCLC largely depends on providers not only keeping abreast current and emerging biomarkers, therapies, and clinical trials but also executing comprehensive testing strategies and practicing good tissue stewardship. Undeniably instrumental in the early days of targeted therapy, SGT is increasingly becoming outdated in the setting of advanced NSCLC due to a number of reasons: 1) an increasing number of treatment targets, 2) inherent limitations of FISH and PCR-based single gene sequencing to detect all relevant alterations, and 3) the acceptance of CGP as a cost-effective and more efficient alternative. Regarding this last point, multiple studies have shown that CGP is a better value because it has improved efficacy for identifying effective treatments, with minimal to no additional cost to patients and payers, despite the up-front price of a CGP testing instance being higher than that of an individual SGTs (10–13). Moreover, guideline-recommended biomarkers such as *ERBB2* mutations, *MET* exon 14 skipping alterations, and *NTRK* fusions are poorly assessed by SGT.

In Case #1, SGT failed to detect the presence of two *ALK* fusions (*EML4-ALK* and *ALK-MAP4K3*), which are the most likely drivers of this tumor’s growth. Importantly, patients with *ALK* fusion-positive NSCLC respond well to ALK-targeting therapies and have meaningful improvements in progression-free and overall survival when treated with ALK inhibitors even at advanced stages (14). DNA-based SGT, such as via PCR and FISH, are capable of identifying many fusion constructs. However, FISH break-apart probes are limited in their ability to detect certain complex or cryptic rearrangements that do not result in detectable probe signals. Importantly, CGP surmounts this limitation, and some platforms, such as OmniSeq INSIGHT, incorporate RNA-based sequencing, specifically for the detection of novel gene fusions (15). This case also raises an important consideration regarding the efficacy of immunotherapy in the setting of certain targetable alterations. For instance, treatment of *ALK* fusion-positive NSCLC is associated with low response rates to immune checkpoint inhibitors (ICIs) and should be avoided in these patients (16). Thus, SGT alone would have missed a targetable alteration and may have prompted a sub-optimal therapy with ICIs.

Case #2 highlights a notable limitation of commercially available PCR-based assays, which may miss as many as 50% of *EGFR* exon 20 insertions, a critical mutation found in up to 12% of NSCLC tumors with *EGFR* mutations that accounts for roughly 4% of all NSCLC cases (17, 18). Exon 20 insertions are highly variable in length and location, often introducing complex secondary structures or occurring in low-complexity regions, complicating probe or primer design in traditional SGT and leading to missed detections of these less common or structurally challenging alterations. NSCLCs driven by *EGFR* exon 20 insertions currently represent a unique treatment situation, since most variants do not respond to 1^st^, 2^nd^, or 3^rd^ generation EGFR TKIs, and first-line therapy options include chemoimmunotherapy or combined amivantamab and systemic chemotherapy (19). In general, identifying *EGFR* mutations early is crucial, as targeted therapies for patients with stage IB-IIIA disease with *EGFR* exon 19 and L858R mutations significantly improve outcomes (20). In case #2, CGP was obtained ~4 months after the initial biopsy, and although the patient’s disease status at the time is not fully known to us, PD-L1 positivity was confirmed before CGP. In the advanced disease setting, such a delay might be concerning because immunotherapy in the presence of oncogenic driver mutations may not be effective or may even be harmful (21). For example, combining EGFR-targeting tyrosine kinase inhibitors with nivolumab increases the risk of pulmonary toxicity (22), and, like *ALK*-rearranged tumors, *EGFR*-mutated tumors generally show poor responses to immune checkpoint inhibitors (ICIs) (16, 23, 24). The effectiveness of ICIs in patients with NSCLC harboring *EGFR* exon 20 insertions remains underexplored, potentially due to a lower prevalence and the aforementioned detection challenges. However, some evidence suggests these patients may not see a survival benefit from ICIs (25). NCCN guidelines advise against PD-1/PD-L1 inhibitors for patients with canonical *EGFR* mutations, such as exon 19 deletion and L858R mutation, as well as *ALK* rearrangements due to limited benefits (19).

CGP provides a significant practical advantage by serving as a comprehensive diagnostic platform, enabling identification of a wide and potentially complete spectrum of targetable genomic alterations. This approach eliminates the necessity of extensive order sets or selection of multiple individual genes from a predetermined list, streamlining the molecular profiling process in clinical settings. This benefit is illustrated in Case #3, in which FISH for *MET* amplification was ordered and interpreted to be negative for amplification, yet CGP identified a targetable *MET* exon 14 skipping alteration. The SGT findings in this case are of uncertain clinical significance: (1) a subset of cells were positive for loss of *MET* and (2) another subset exhibited additional copies of *MET* and the control probe, consistent with polysomy for chromosome 7. Importantly, FISH for *MET* amplification is not capable of uncovering actionable single nucleotide variants of *MET*. In contrast, CGP identified a *MET* exon 14 skipping alteration, and targeting this alteration with *MET*-specific TKIs (such as tepotinib or capmatinib) can significantly improve clinical outcomes (26–29). It is also worth noting that CGP was ordered much later than SGT (~7-8 months after SGT), suggesting that the patient was initially diagnosed with an early stage cancer that progressed.

Case #4 illustrates another practical advantage of CGP over SGT. In this case, CGP identified a *BRAF* V600E mutation, a targetable tumor-agnostic biomarker, but, to our knowledge, SGT was not ordered to evaluate this specific variant. In contrast to SGT, CGP platforms typically can evaluate many tumor agnostic biomarkers, including *BRAF* V600E, MMR deficiency/MSI status, TMB assessment, *NTRK* fusions, and *RET* fusions. This comprehensive, single-assay approach can not only alleviate logistical burdens and enhance diagnostic efficiency for clinicians, but it can also conserve tissue for other tests (such as IHC) and prevent the need for costly re-sampling procedures. The issue of tissue stewardship deserves careful consideration in NSCLC management because the extensive tissue sections required for completing all necessary SGTs often exceed those needed for NGS, making CGP a more tissue-conservative option (5). This consideration is particularly important in lung cancer, which frequently yields limited biopsy samples.

As a reference laboratory, there are limitations with our reporting of this case series. For instance, we do not have knowledge of all testing platforms that patients received, their therapeutic regimens, their tumor stage at diagnosis, or their outcomes. Despite these shortcomings, we can make a number of important observations related to the consequences of SGT. First, we can confirm that multiple SGTs were performed in each case, which increases the risk of exhausting tissue for additional testing, and 75% (3/4) of these cases used the same tissue block for SGT and CGP. Second, from the results and indications for testing, we can deduce that SGT alone would have missed actionable variants for biomarker-directed therapy in the advanced stage setting. Third, each of these cases highlights the need for providers to consider CGP to test a broad array of variants to better inform the treatment of NSCLC. Fourth, the turnaround time for CGP in all 4 cases was less than or equal to the turnaround time (TAT) for all SGTs to be reported, contesting the generally accepted proposition that TAT is greatly shorter for SGT than CGP. Although SGT may be preferred in select clinical settings, its inherent limitations will result in missed therapeutic opportunities in the aggregate. CGP offers the benefits of comprehensive testing for novel fusions, detecting uncommon and complex actionable variants, and reducing clinician burden through simplification of testing. Of note,

As our understanding of cancer genomics continues to evolve and as costs associated with CGP continue to decrease, it is expected that the utility of CGP in guiding personalized therapeutic strategies for NSCLC will further consolidate, resulting in more cost-effective and time-efficient results. However, the adoption of CGP is not without challenges. Significant barriers to widespread implementation include a perception of high costs, the need for advanced sequencing machinery, the requirements of specialized bioinformatics support to interpret complex data outputs, and low rates of reimbursement. Additionally, the clinical significance of some genetic alterations identified by CGP may not be fully understood yet, necessitating scientific research, continual education, and regular collaboration between oncologists, pathologists, and geneticists to translate these findings into actionable clinical strategies. Despite these challenges, the potential of CGP to enhance personalized cancer care and improve patient outcomes justifies its increasing role in the management of NSCLC.

## Patient perspective

Patients facing conflicting test results or learning that a different testing method could have influenced their treatment options may experience a range of emotions, ranging from confusion and frustration to warranted concern about the implications for their care. For instance, in Case #1, in which SGT failed to detect ALK fusions later identified by CGP, a patient might feel both relief that a more effective treatment option is available but also anxiety about the delay in receiving this targeted therapy. The situation may feel even more dire for patients who receive sub-optimal therapies (such as ICIs in the setting of tumors with *ALK* or *EGFR* alterations). The realization that earlier comprehensive testing could have impacted their course of therapy could damage the physician-patient relationship.

Delays in ordering CGP, as potentially seen in Case #2, could lead to feelings of missed opportunity, especially when considering the potential benefits of a therapy specifically targeted to their mutation. Patients might question the rationale behind the initial choice of SGT and its impact on their treatment timeline and overall prognosis, knowing that a more complete solution was available in a single test. If healthcare providers insist on ordering SGT, there must be clear communication with patients about the rationale for the specific test, the possibility of missed therapeutic options, and the potential need for subsequent tests based on the results and evolving clinical insights.

From the patient’s perspective, understanding the technical differences between SGT and CGP might be challenging, but grasping the practical implications—such as the potential for more effective, personalized treatment options—is crucial. Patients may also appreciate learning about the concept of tissue stewardship, especially in cases where limited biopsy material necessitates careful consideration of the most informative and efficient testing strategy.

Ultimately, patients deserve assurance that their treatment is based on the most accurate and comprehensive information available. Conflicting test results or the knowledge that different testing could influence therapy may prompt patients to advocate for themselves more strongly, seeking second opinions or request CGP to ensure that their treatment plan is as effective as possible.

## Data Availability

The original contributions presented in the study are included in the article/**Supplementary Material**. Further inquiries can be directed to the corresponding author.

## References

[1] Ferreira CG Reis MX Veloso GGV. Editorial: Molecular genetic testing and emerging targeted therapies for non-small cell lung cancer. Front Oncol. (2023) 13. doi: 10.3389/fonc.2023.1308525 PMC10704016 38074696

[2] Alduais Y Zhang H Fan F Chen J Chen B. Non-small cell lung cancer (NSCLC): A review of risk factors, diagnosis, and treatment. Med (Baltimore). (2023) 102:e32899. doi: 10.1097/MD.0000000000032899 PMC11309591 36827002

[3] Shen T Pajaro-Van De Stadt SH Yeat NC Lin JCH. Clinical applications of next generation sequencing in cancer: from panels, to exomes, to genomes. Front Genet. (2015) 6. doi: 10.3389/fgene.2015.00215 PMC4469892 26136771

[4] Lemmon CA Zhou J Hobbs B Pennell NA. Modeling costs and life-years gained by population-wide next-generation sequencing or single-gene testing in nonsquamous non–small-cell lung cancer in the United States. JCO Precis Oncol. (2023) 7. doi: 10.1200/PO.22.00294 PMC9928881 36634300

[5] Nesline MK Subbiah V Previs RA Strickland KC Ko H Depietro P. The impact of prior single-gene testing on comprehensive genomic profiling results for patients with non-small cell lung cancer. Oncol Ther. (2024) 12(2):329–343. doi: 10.1007/s40487-024-00270-x PMC11187032 38502426

[6] Shen C-I Chiang C-L Shiao T-H Luo Y-H Chao H-S Huang H-C. Real-world evidence of the intrinsic limitations of PCR-based EGFR mutation assay in non-small cell lung cancer. Sci Rep. (2022) 12. doi: 10.1038/s41598-022-17394-7 PMC9363455 35945330

[7] Tuononen K Mäki-Nevala S Sarhadi VK Wirtanen A Rönty M Salmenkivi K. Comparison of targeted next-generation sequencing (NGS) and real-time PCR in the detection of *EGFR*, *KRAS*, and *BRAF* mutations on formalin-fixed, paraffin-embedded tumor material of non-small cell lung carcinoma—Superiority of NGS. Genes Chromosomes Cancer. (2013) 52:503–11. doi: 10.1002/gcc.22047 23362162

[8] Herbst RS Aisner DL Sonett JR Turk AT Weintraub JL Lindeman NI. Practical considerations relating to routine clinical biomarker testing for non–small cell lung cancer: focus on testing for RET fusions. Front Med. (2021) 7. doi: 10.3389/fmed.2020.562480 PMC7859651 33553195

[9] Conroy JM Pabla S Glenn ST Seager RJ Van Roey E Gao S. A scalable high-throughput targeted next-generation sequencing assay for comprehensive genomic profiling of solid tumors. PloS One. (2021) 16:e0260089. doi: 10.1371/journal.pone.0260089 34855780 PMC8639101

[10] Pennell NA Mutebi A Zhou ZY Ricculli ML Tang W Wang H. Economic impact of next-generation sequencing versus single-gene testing to detect genomic alterations in metastatic non-small-cell lung cancer using a decision analytic model. JCO Precis Oncol. (2019) 3:1–9. doi: 10.1200/PO.18.00356 35100695

[11] Vanderpoel J Stevens AL Emond B Lafeuille M-H Hilts A Lefebvre P. Total cost of testing for genomic alterations associated with next-generation sequencing versus polymerase chain reaction testing strategies among patients with metastatic non-small cell lung cancer. J Med Econ. (2022) 25:457–68. doi: 10.1080/13696998.2022.2053403 35289703

[12] Wong W Sheinson D Liu Y To TM. Costs associated with the use of multigene panel tests in three solid tumor types and the impact on insurance premiums. Future Oncol. (2023) 19:705–14. doi: 10.2217/fon-2023-0094 37070428

[13] Harvey MJ Cunningham R Sawchyn B Montesion M Reddy P McBride A. Budget impact analysis of comprehensive genomic profiling in patients with advanced non–small-cell lung cancer. JCO Precis Oncol. (2021) 5:1611–24. doi: 10.1200/PO.20.00540 34994647

[14] Mok T Camidge DR Gadgeel SM Rosell R Dziadziuszko R Kim DW. Updated overall survival and final progression-free survival data for patients with treatment-naive advanced ALK-positive non-small-cell lung cancer in the ALEX study. Ann Oncol. (2020) 31:1056–64. doi: 10.1016/j.annonc.2020.04.478 32418886

[15] Severson E Achyut BR Nesline M Pabla S Previs RA Kannan G. RNA sequencing identifies novel NRG1 fusions in solid tumors that lack co-occurring oncogenic drivers. J Mol Diagn. (2023) 25:454–66. doi: 10.1016/j.jmoldx.2023.03.011 37164276

[16] Gainor JF Shaw AT Sequist LV Fu X Azzoli CG Piotrowska Z. *EGFR* mutations and *ALK* rearrangements are associated with low response rates to PD-1 pathway blockade in non–small cell lung cancer: A retrospective analysis. Clin Cancer Res. (2016) 22:4585–93. doi: 10.1158/1078-0432.CCR-15-3101 PMC5026567 27225694

[17] Viteri S Minchom A Bazhenova L Ou SHI Bauml JM Shell SA. Frequency, underdiagnosis, and heterogeneity of epidermal growth factor receptor exon 20 insertion mutations using real-world genomic datasets. Mol Oncol. (2023) 17:230–7. doi: 10.1002/1878-0261.13327 PMC9892822 36269676

[18] Burnett H Emich H Carroll C Stapleton N Mahadevia P Li T. Epidemiological and clinical burden of EGFR Exon 20 insertion in advanced non-small cell lung cancer: A systematic literature review. PloS One. (2021) 16:e0247620. doi: 10.1371/journal.pone.0247620 33684140 PMC7939356

[19] Ettinger DS Wood DE Aisner DL Akerley W Bauman JR Bharat A. NCCN guidelines^®^ Insights: non–small cell lung cancer, version 2.2023. J Natl Compr Cancer Network. (2023) 21:340–50. doi: 10.6004/jnccn.2023.0020 37015337

[20] Herbst RS Wu YL John T Grohe C Majem M Wang J. Adjuvant osimertinib for resected EGFR-mutated stage IB-IIIA non-small-cell lung cancer: updated results from the phase III randomized ADAURA trial. J Clin Oncol. (2023) 41:1830–40. doi: 10.1200/JCO.22.02186 PMC10082285 36720083

[21] Kalra A Rashdan S. The toxicity associated with combining immune check point inhibitors with tyrosine kinase inhibitors in patients with non-small cell lung cancer. Front Oncol. (2023) 13. doi: 10.3389/fonc.2023.1158417 PMC10140561 37124513

[22] Oshima Y Tanimoto T Yuji K Tojo A. EGFR–TKI-associated interstitial pneumonitis in nivolumab-treated patients with non–small cell lung cancer. JAMA Oncol. (2018) 4:1112. doi: 10.1001/jamaoncol.2017.4526 29327061 PMC5885195

[23] To KKW Fong W Cho WCS. Immunotherapy in treating EGFR-mutant lung cancer: current challenges and new strategies. Front Oncol. (2021) 11. doi: 10.3389/fonc.2021.635007 PMC8185359 34113560

[24] Lee CK Man J Lord S Links M Gebski V Mok T. Checkpoint inhibitors in metastatic EGFR- mutated non–small cell lung cancer—A meta-analysis. J Thorac Oncol. (2017) 12:403–7. doi: 10.1016/j.jtho.2016.10.007 27765535

[25] Yang G Yang Y Liu R Li W Xu H Hao X. First-line immunotherapy or angiogenesis inhibitor combined with chemotherapy for advanced non-small cell lung cancer with *EGFR* exon 20 insertions: Real-world evidence from China. Cancer Med. (2023) 12:335–44. doi: 10.1002/cam4.v12.1 PMC9844624 35608132

[26] Le X Sakai H Felip E Veillon R Garassino MC Raskin J. Tepotinib efficacy and safety in patients with MET exon 14 skipping NSCLC: outcomes in patient subgroups from the VISION study with relevance for clinical practice. Clin Cancer Res. (2022) 28:1117–26. doi: 10.1158/1078-0432.CCR-21-2733 PMC9365370 34789481

[27] Mathieu LN Larkins E Akinboro O Roy P Amatya AK Fiero MH. FDA approval summary: capmatinib and tepotinib for the treatment of metastatic NSCLC harboring MET exon 14 skipping mutations or alterations. Clin Cancer Res. (2022) 28:249–54. doi: 10.1158/1078-0432.CCR-21-1566 34344795

[28] Mazieres J Paik PK Garassino MC Le X Sakai H Veillon R. Tepotinib treatment in patients with MET exon 14-skipping non-small cell lung cancer: long-term follow-up of the VISION phase 2 nonrandomized clinical trial. JAMA Oncol. (2023). doi: 10.1001/jamaoncol.2023.1962 PMC10240398 37270698

[29] Wolf J Garon EB Groen HJ Tan DS-W Robeva A Le Mouhaer S. Capmatinib in MET exon 14-mutated, advanced NSCLC: Updated results from the GEOMETRY mono-1 study. J Clin Oncol. (2021) 39:9020. doi: 10.1200/JCO.2021.39.15_suppl.9020

